# Quantitative Analysis of Selected Circulating Hematological Biomarkers, Essential Minerals, Vitamins, and Thyroid Hormones in Females Affected by Hair Loss

**DOI:** 10.3390/diseases13110352

**Published:** 2025-10-29

**Authors:** Saad Al-Fawaeir, Ibrahim Al-Odat

**Affiliations:** Department of Medical Laboratory Science, Faculty of Allied Medical Sciences, Jadara University, Irbid 21110, Jordan; s.alfawaeir@jadara.edu.jo

**Keywords:** hair loss, minerals, thyroid hormones, vitamins, women health

## Abstract

Purpose: To assess the association between hair loss in females and various biomarkers including hemoglobin, iron, ferritin, zinc, selenium, calcium, vitamin D, vitamin B12, folic acid, and thyroid hormones. Patients and methods: This study enrolled 100 women presenting with hair loss and 100 age-matched healthy controls. Venous blood samples were collected for analysis of hematological, hormonal and biochemical parameters. Results: The mean age of participants was comparable between groups (43.06 ± 10.76 vs. 41.39 ± 7.94 years; *p* = 0.88). Hair loss in females had significantly lower mean levels of Hb (11.45 ± 0.39 vs. 13.09 ± 0.46 g/dL; *p* < 0.001), iron (70.14 ± 7.85 vs. 94.42 ± 5.61 µg/dL; *p* < 0.001) and ferritin (39.34 ± 3.71 vs. 48.09 ± 5.31 ng/mL), all with *p* < 0.001. Serum levels of selenium (67.11 ± 5.53 vs. 71.45 ± 4.05 µg/L), zinc (86.07 ± 3.98 vs. 88.87 ± 2.03 µg/L), copper (90.71 ± 3.48 vs. 104.84 ± 5.38 µg/L), and calcium (8.61 ± 0.28 vs. 9.11 ± 0.27 mg/dL) were significantly reduced in women with hair loss (*p* < 0.001). Thyroid hormones were also significantly lower in the hair loss group, including TSH (1.74 ± 0.25 vs. 2.35 ± 0.39 µIU/mL) and FREE T4 (1.11 ± 0.11 vs. 1.32 ± 0.12 ng/dL), despite remaining within the normal reference ranges. Patients also showed lower serum folate (6.17 ± 0.63 vs. 6.96 ± 0.41 ng/mL), vitamin B12 (185.52 ± 35.27 vs. 258.30 ± 52.84 pg/mL), and vitamin D (26.32 ± 2.98 vs. 32.20 ± 3.76 ng/dL) levels (*p* < 0.001). Conclusions: Hair loss in females is significantly associated with reduced levels of circulating hemoglobin, iron, copper, selenium, vitamin D, vitamin B12, folate, thyroid-stimulating hormone and FREE T4 hormone.

## 1. Introduction

Hair loss, also referred to as alopecia, is a disorder resulting from a disruption in the normal cycle of hair production [1]. It can affect individuals at various stages of life and excessive hair shedding affects a high proportion of individuals worldwide. By the time they are 50 years old, more than 25% of women are predicted to have female-pattern hair loss (FPHL) [2], while male-pattern hair loss impacts up to 80% of men and 50% of women over their lifetimes [3]. Hair loss tends to have a greater psychological impact on females compared to males [4]. Generally, it is associated with a wide range of contributing factors including hormonal imbalances, nutritional deficiencies, stress experiencing, chronic illnesses, and lifestyle-related elements that adversely affect hair growth and follicular health [5]. These factors directly influence the hair growth cycle and contribute to weakening of hair roots [6].

In women, various types of alopecia exist, with telogen effluvium, characterized by excessive hair shedding during the telogen (resting) phase, being the most common [7]. Hair loss may be caused by multiple triggers such as thyroid disorders, acute blood loss, poor nutrition, and malnutrition [8]. Therefore, nutrients, such as trace minerals and vitamins, are critical dietary components required for maintaining normal hair physiology [9]. Vitamin D (25-hydroxyvitamin D3) contributes significantly to a variety of cellular functions, particularly the function of skin and hair follicle cells [10].

Four phases make up the ongoing process of hair growth: anagen (growth), catagen (regression), telogen, and exogen (shedding). Each hair follicle undergoes 10 to 30 cycles throughout its lifespan [11]. During the telogen phase, approximately 10–15% of the scalp’s hair (roughly 100,000 strands) enters a resting state, and it is normal to shed between 100 and 150 hairs per day. Hair shedding during the anagen phase is rare [11]. The vitamin D receptor (VDR) plays a pivotal role in stimulating follicular activity during the anagen phase [12], and a deficiency in vitamin D has been linked to impaired hair follicle growth and increased shedding [13].

Trace elements such as zinc (Zn^2+^) are essential for numerous biological processes. Zinc is a cofactor in many enzymatic reactions and plays a functional role in hair follicles by promoting repair and inhibiting regression. Additionally, it helps maintain the health of the sebaceous glands associated with hair follicles [14,15]. Copper (Cu) also contributes to hair health through its involvement in hemoglobin synthesis, which is vital for ensuring adequate oxygen transport to hair shafts. Copper deficiency may impair shaft structure and contribute to increased hair loss [16].

Iron (Fe) deficiency also plays a substantial role in hair loss. Low iron stores, particularly serum ferritin, along with reduced levels of hemoglobin and vitamin B12, are frequently implicated. As a result, routine laboratory evaluation of ferritin, hemoglobin, and vitamin B12 levels is recommended in dermatological practice to screen for underlying nutritional deficiencies contributing to hair loss [17].

Thyroid dysfunction is another major endocrine-related cause of hair loss. Triiodothyronine (T3) and thyroxine (T4) are two physiologically active compounds secreted by the thyroid gland which are essential for cellular growth, differentiation, metabolism, and the regulation of skin and hair follicle turnover. The skin is a major target organ for thyroid hormones. Diffuse hair loss is commonly observed in patients with hypothyroidism, hyperthyroidism, or drug-induced thyroid abnormalities. Notably, hair loss is reported in approximately 33% of individuals with hypothyroidism and 50% of those with hyperthyroidism [18]. In hypothyroidism, elevated telogen rates can lead to noticeable changes in hair properties, including increased dryness, brittleness, and coarseness. These changes are accompanied by reduced proliferation of hair bulb cells, resulting in progressive hair thinning [13].

This study addresses a critical gap in the existing literature by comprehensively evaluating a broad range of biochemical, hematological, and hormonal parameters associated with hair loss in women, a multifactorial condition often underexplored in its systemic context. Unlike previous studies that typically focus on a limited set of markers, this research uniquely integrates micronutrients (such as selenium, zinc, copper), vitamins (D, B12, folate), iron indices, calcium, and thyroid hormones, using a robust comparative design with age-matched controls. Conducted on a Jordanian female population, it also adds regional specificity, which is rarely represented in global data. The study’s findings offer clinicians a more holistic understanding of underlying deficiencies that may contribute to hair loss, highlighting potential targets for personalized treatment strategies and preventative care.

Investigating the relationships between hair loss and other biochemical indicators, such as hemoglobin (Hb), selenium, zinc, calcium, iron, ferritin, folic acid, and thyroid hormones in a representative sample of Jordanian women, is the goal of the current study.

## 2. Materials and Methods

### 2.1. Ethical Approval

Ethical approval for this study was obtained from the relevant institutional ethics review board and filed under JA/AMS/3_2025. All procedures were performed in accordance with the principles of the Declaration of Helsinki.

### 2.2. Study Design and Participants

This prospective case–control study was conducted at multiple private dermatology clinics located in Al-Salt City, Jordan. Data collection took place over 2 months period, from March 2025 to April 2025.

A total of 200 female participants were voluntarily enrolled in this research work, with 100 patients presenting with hair loss and 100 age-matched healthy women with no hair loss. The goal and methods of the investigation were clearly explained to each participant and signed informed consent form were obtained from all participants. Participants’ privacy and confidentiality were always maintained.

To ensure the validity of the biochemical measurements, all participants were screened for underlying conditions that could influence the parameters under investigation.

### 2.3. Inclusion and Exclusion Criteria

Only females experiencing hair loss were included in the experimental group. All patients presented with diffuse, non-scarring alopecia clinically consistent with telogen effluvium. The diagnosis was established through clinical history, physical examination, dermoscopic evaluation, and hair pull test. Participants were excluded if they were taking nutritional supplements or had systemic illnesses, infections, or positive C-reactive protein levels, as these factors could influence serum ferritin levels. Additional exclusion criteria included chronic conditions such as liver disease, diabetes, anemia from non-iron deficiency causes, endocrine disorders, and current chemotherapy treatment. Patients diagnosed with androgenic alopecia, alopecia areata, cicatricial alopecia, or any other forms of pathological hair loss were also excluded on the basis of characteristic clinical and dermoscopic findings. The diagnosis and exclusion were performed by a board-certified dermatologist. Only demographic (age, sex) and biochemical variables were collected.

### 2.4. Blood Samples Collection

All participants had a venous blood specimens taken between 8:00 and 10:00 AM in separate test tubes. For hematological assessments, samples were collected in EDTA tubes. Blood was extracted into gel-separator vials for biochemical testing, left to coagulate at room temperature for fifteen minutes, and then centrifuged for ten minutes at 4000× *g*. Serum samples were allocated into Eppendorf tubes and immediately analyzed.

### 2.5. Laboratory Measurements

The biochemical tests conducted included Hb, thyroid stimulating hormone (TSH) and free T4, vitamin D3, vitamin B12, folate, serum iron, ferritin, calcium, zinc, selenium, and copper. Thyroid hormones, vitamin D3, folate, and ferritin were measured using the Cobas e411 automated analyzer (Roche Diagnostics, Mannheim, Germany), a fully automated immunoassay system. Serum concentrations of iron, calcium, copper, selenium, and zinc were assessed using the Cobas c111 chemistry analyzer (Roche Diagnostics, Mannheim, Germany). Hematological parameters, including Hb and complete blood counts, were determined using the Sysmex K1000 automated hematology analyzer (Tao Electronics, Aichi, Japan), with regular calibration and quality control procedures in place.

### 2.6. Reference Ranges

The reference values used in the study were as follows:

TSH: 0.4–4.2 µIU/mL, FT4: 0.7–1.7 ng/dL, Hb: 11.6–15 g/dL, Zn: 70–110 µg/L, Cu: 70–140 µg/L, Folate: 3–17 ng/mL, Vitamin B12: 160–950 pg/mL, Vitamin D: 30–100 ng/dL, Ca: 8.4–10 mg/dL, Fe: 70–120 µg/dL, ferritin: 13 to 150 ng/mL.

### 2.7. Statistical Analysis

The means ± standard deviations (SD) were used to express the data. Microsoft Excel 365 was used for the means and standard deviations calculations while SPSS software version 20.0 (SPSS Inc., Chicago, IL, USA) was used for statistical analysis. Statistical comparisons were conducted using chi-square tests, one-way ANOVA, and multivariable binary logistic regression. *p*-values below 0.05 were regarded as statistically significant.

## 3. Results

### 3.1. Age Distribution Between Female Hair Loss Patients and Controls

The mean age of the women with hair loss was 43.06 ± 10.76 years, while that of the control group was 41.39 ± 7.94 years (Table 1). The age range was similar between groups, with patients ranging from 26 to 65 years and controls from 26 to 59 years. The distribution of participants under and over 45 years was also comparable, with 56% of hair loss patients and 58% of controls being under 45 years of age. Statistical analysis using the chi-square test yielded a test value of 0.08 and a *p*-value of 0.88, indicating no statistically significant difference in age distribution between the two groups.

### 3.2. Comparison of Hematological Parameters Between Female with Hair Loss and Controls

Hb levels were significantly reduced in women with hair loss (11.45 ± 0.39 g/dL) compared to controls (13.09 ± 0.46 g/dL) (Table 2), the mean Hb in hair loss patients (11.45 g/dL) was slightly below the lower reference limit, consistent with borderline anemia, while controls remained within normal. Similarly, serum iron levels were markedly reduced in women with hair loss (70.14 ± 7.85 µg/dL) compared to controls (94.42 ± 5.61 µg/dL), approaching the lower limit of the normal range (70 to 120 µg/dL). Ferritin levels also showed a significant difference, with women with hair loss recording a mean of 39.34 ± 3.71 ng/mL versus 48.09 ± 5.31 ng/mL in controls, though both values remained within the reference range (13 to 150 ng/mL). All differences were statistically significant with *p*-values < 0.001, demonstrating a robust linkage between lower hematological indices and hair loss in females.

### 3.3. Comparison of Serum Trace Elements and Calcium Levels Between Female with Hair Loss and Controls

Table 3 represents a comparison of serum selenium, zinc, copper, and calcium levels between 100 females with hair loss and 100 healthy female controls. The mean selenium level was significantly lower in the patient group (67.11 ± 5.53 µg/L) compared to controls (71.45 ± 4.05 µg/L), with an f-value of 40.04 and a *p*-value < 0.001. Similarly, zinc levels were reduced in patients (86.07 ± 3.98 µg/L) versus controls (88.87 ± 2.03 µg/L), though both means remained within the normal range (70–110 µg/L). Copper levels showed a pronounced difference, with patients exhibiting a lower mean (90.71 ± 3.48 µg/L) compared to controls (104.84 ± 5.38 µg/L), well within the reference range (70–140 µg/L). Serum calcium was also significantly reduced in the hair loss group (8.61 ± 0.28 mg/dL) relative to controls (9.11 ± 0.27 mg/dL), although both averages remained within the normal physiological range (8.4–10 mg/dL). All comparisons revealed statistically significant differences, with *p*-values less than 0.001, suggesting a potential link between hair loss and reduced serum levels of these micronutrients in females.

### 3.4. Comparison of Thyroid Function Parameters Between Female with Hair Loss and Controls

Table 4 illustrates a comparison of thyroid function markers (TSH and FREE T4) between 100 females with hair loss and 100 healthy female controls. The mean serum TSH level in the patient group was significantly lower (1.74 ± 0.25 µIU/mL) than in the control group (2.35 ± 0.39 µIU/mL), although both values were within the normal reference range of 0.4 to 4.2 µIU/mL. Similarly, the mean FREE T4 level was also significantly reduced in females with hair loss (1.11 ± 0.11 ng/dL) compared to controls (1.32 ± 0.12 ng/dL), with both means falling within the normal range of 0.7 to 1.7 ng/dL. Statistical analysis demonstrated highly significant differences for both parameters, with f-values of 169.91 and 170.64 for TSH and FREE T4, respectively, and *p*-values < 0.001. These findings suggest that altered, yet still clinically normal, thyroid hormone levels may be associated with hair loss in females.

### 3.5. Comparison of Serum Folate, Vitamin B12, and Vitamin D Levels Between Female with Hair Loss and Controls

Table 5 presents a comparison of serum folate, vitamin B12, and vitamin D levels between 100 female hair loss patients and 100 healthy female controls. Females with hair loss had significantly lower folate levels (6.17 ± 0.63 ng/mL) compared to controls (6.96 ± 0.41 ng/mL), with both means within the normal range of 3 to 17 ng/mL. Vitamin B12 levels were also significantly reduced in patients (185.52 ± 35.27 pg/mL) relative to controls (258.30 ± 52.84 pg/mL), though all values remained above the lower limit of the reference range (160 to 950 pg/mL). Notably, serum vitamin D levels in females with hair loss (26.32 ± 2.98 ng/dL) were below the normal threshold (30 to 100 ng/dL), in contrast to controls who had a mean level of 32.20 ± 3.76 ng/dL. All differences were statistically significant with *p*-values < 0.001 and high f-values, suggesting a strong association between reduced micronutrient levels and hair loss in females.

### 3.6. Analysis of Biochemical Predictors Associated with Hair Loss in Females

Table 6 presents the results of multivariable logistic regression analysis of predictors of hair loss in females. In the multivariable logistic regression model, several factors were identified as significant predictors of hair loss. Younger age was independently associated with an increased likelihood of hair loss (OR = 0.94, 95% CI: 0.89–0.98, *p* = 0.008). Lower serum selenium (OR = 0.62, 95% CI: 0.38–0.98, *p* = 0.041), lower TSH (OR = 0.44, 95% CI: 0.22–0.90, *p* = 0.025), reduced folate (OR = 0.79, 95% CI: 0.63–0.99, *p* = 0.045), lower vitamin B12 (OR = 0.99, 95% CI: 0.98–0.99, *p* = 0.012), lower vitamin D (OR = 0.94, 95% CI: 0.89–0.99, *p* = 0.021), and lower ferritin (OR = 0.96, 95% CI: 0.93–0.99, *p* = 0.033) were also significantly associated with hair loss. In contrast, hemoglobin, zinc, copper, calcium, iron, and FREE T4 levels were not significantly related to hair loss in the adjusted model. The model showed acceptable calibration (Hosmer–Lemeshow test *p* = 0.47) and moderate explanatory power (Nagelkerke R^2^ = 0.36).

## 4. Discussion

The key finding of our study underscores several important predictors and contributing factors to hair loss in women, particularly hematological, biochemical, and hormonal biomarkers. Notably, hair loss-affected women exhibited lower levels of hemoglobin, iron, ferritin, vitamin D, vitamin B12, and TSH.

For women, hair loss is a debilitating condition. It can affect female’s sense of self-esteem and be linked to a negative body image impression [19]. Prior to seeking dermatological consultation, patients typically try various treatment methods (such as shampoos, nutritional supplements, and medical treatment) in hopes of promoting hair regrowth [20]. Consequently, the initial dermatological consultation session is often delayed after the onset of hair loss. This delay may contribute to poor adherence and results, particularly if female have irrational expectations for the course of therapy [20]. Biomarkers are crucial for understanding the pathophysiology, activity, and degree of hair loss. Numerous studies have identified and confirmed several biomarkers associated with hair loss [21,22,23].

In the current study, Hb levels were significantly lower in women with hair loss compared to controls. In alignment with this finding, a Jordanian retrospective study reported that Hb levels were also considerably lower in women affected by hair loss (12.8 ± 1.3 g/dL) compared to the placebo group (13.4 ± 1.3 g/dL), (*p* < 0.05) [21]. Similarly, other studies found a significant decrease in Hb levels in people affected by hair loss (11.18 g/dL) compared to healthy people (13.2 g/dL; *p*-value < 0.05) [24]. Nasrin et al. (2024) also observed similar findings and reported that 66.7% of FPHL patients had low Hb levels (<12 µg/L) with a mean of 11.47 ± 1.52 µg/L [20].

In contrast, Mohammed et al. (2024) found no significant differences in Hb levels between alopecia patients from Iraq and healthy individuals [25]. Hegde and Noronha (2020) also found that the average Hb level in patients was 12.41 ± 1.31 g/dL compared to 12.84 ± 1.08 g/dL in healthy participants [26]. The Hb levels of the patients and healthy people did not differ statistically significantly. Furthermore, Elethawi and Jabbar (2012) reported that level of Hb among hair loss patients was 12.37 g/dL compared to 12.19 g/dL in controls [27]. Hb levels remain within normal limits in women with hair loss.

Hb levels decreased in females with hair loss may be due to underlying iron deficiency, which is a common cause of both anemia and hair shedding. Iron is essential for hemoglobin synthesis and also plays a critical role in the proliferation of hair follicle cells. Inadequate iron stores can impair oxygen transport to rapidly dividing cells in the hair matrix, leading to weakened hair structure and increased hair loss. Additionally, factors such as menstrual blood loss, poor dietary intake, or malabsorption can contribute to lower Hb levels in women, further exacerbating hair thinning [28].

Serum ferritin levels are directly correlate with the body’s iron status [29] and decreased ferritin levels means iron deficiency [30]. In the current study, we observed that serum iron and ferritin levels were significantly reduced in women affected by hair loss compared to the control group. Nevertheless, other studies contradicted our findings by reporting no significant difference in serum iron levels between individuals with hair loss and healthy controls [24,26].

In accordance with our finding, Kantor et al. reported that alopecia in females is linked to low serum ferritin levels [31]. Moreover, several studies found that patients with FPHL had a significantly lower level of serum ferritin [32]. Nasrin et al. (2024) also found that approximately 86.7% of FPHL patients had ferritin levels below 30 ng/mL), with a mean of 20.25 ± 16.07 ng/mL [20]. Similarly, Siah et al. (2016) demonstrated markedly reduced ferritin levels in girls with FPHL [33]. According to Rasheed et al. (2013), FPHL patients (23.9 ± 38.5 μg/L) and those with telogen effluvium (TE) (14.7 ± 22.1 μg/L) had significantly lower ferritin levels compared to controls (43.5 ± 20.4 μg/L) [34].

Conversely, Hegde & Noronha (2020) found that, among hair loss patients, the mean serum ferritin levels were 37.25 ± 25.74 ng/mL, while among healthy participants were 40.62 ± 32.24 ng/mL [26]. In this regard, there was no statistically significant difference in serum ferritin levels between individuals with hair loss and healthy individuals.

Serum ferritin is a secreted component of glycosylated peptide that often rises in liver illness, inflammation, cancer, or iron overload, and falls when tissue supply of iron is depleted. In the dearth of chronic illnesses or inflammatory conditions, it is a reliable marker of low iron stores [35]. Ferritin is also found in hair follicles; a decrease in ferritin reserves within the follicle impairs hair growth, leading to hair loss [24]. Thus, serum ferritin is a useful biomarker in the assessment of hair loss [24]. Moreover, insufficient ferritin levels have been linked to morphological changes in hair, including dryness, brittleness, inability to retain curl or color, and breakage [36]. Despite this, the role of iron in hair loss remains controversial due to inconsistent findings across studies [37].

In the current study, we also found that serum vitamin D levels in women with hair loss were significantly below both the normal threshold and the levels in the control group (*p* < 0.001). Mohammed et al. (2024) reported similar findings and stated that the mean serum vitamin D levels in hair loss patients and control groups were 18.12 ± 26.20 ng/mL and 41.04 ± 5.54 ng/mL, respectively, with a statistically significant difference (*p* < 0.001) [25]. Farah et al. (2021) also supported these results, reporting that the concentration of serum vitamin D was significantly lower in the hair loss group (28.7 ± 5.8 ng/mL) compared to the control group (34.2 ± 7.1 ng/mL; *p* < 0.05) [21]. Siah et al., also reported a significant reduction in serum vitamin D among females with FPHL compared to standard reference levels [33]. Rasheed et al. (2013) reported that women with FPHL (29.1 ± 8.5 nmol/L) and TE (28.8 ± 10.5 nmol/L) had significantly reduced serum vitamin D2 levels than healthy subjects (118.2 ± 68.1 nmol/L; *p* < 0.001) [34]. Furthermore, Rasheed et al. observed a negative correlation between the severity of the hair loss condition and reduced level of serum vitamin D2. Findings align with Zhao et al. (2020), who demonstrated that vitamin D plays a role in hair follicle cycle and associated disorders and may be utilized as a therapy to promote hair growth [38].

The observed reduction in serum vitamin D levels among patients with hair loss may be explained by the role of vitamin D receptors, which are expressed in hair follicles. Activation of these receptors is essential for initiating the anagen (growth) phase [39]. A deficiency in vitamin D may lead to impaired follicular development and premature entry into the telogen (resting) phase, resulting in increased hair shedding [11]. Additionally, individuals with limited sun exposure, darker skin pigmentation, or poor dietary intake are more prone to vitamin D deficiency, which may be more prevalent among those experiencing hair loss. Chronic inflammation and autoimmune disorders, which are sometimes associated with hair loss conditions like alopecia areata, may also contribute to lower serum vitamin D levels by altering its metabolism or increasing its utilization [40].

In the current study, vitamin B12 levels were significantly reduced in hair loss patients compared to controls; however, all means values remained above the lower limit of the reference range.

This aligns with previous studies reported similar findings. Farah et al. (2021) showed that vitamin B12 levels were significantly decreased in the hair loss group (258.8 ± 75.7 ng/mL) compared to the control group (392.6 ± 41.3 ng/mL; *p* < 0.05) [21]. Additionally, Belgaumkar et al. (2021) stated that 35.7% of females with hair loss had inadequate vitamin B12 levels [41]. Ertug and Yilmaz (2018) also observed significantly lower vitamin B12 levels in the telogen effluvium (TE) group (232.13 ± 123.35 pg/mL; *p* = 0.001) [42].

Conversely, Mohammed et al. (2024) reported no statistically significant difference in vitamin B12 levels between hair loss patients and control group [25]. Likewise, Durusu Turkoglu et al. (2024) found no significant difference in vitamin B12 levels between chronic telogen effluvium (CTE) patients and healthy controls [43].

Vitamin B12 is often reduced in hair loss patients because it plays a vital role in red blood cell formation and DNA synthesis [44], both of which are critical for the maintenance of healthy hair follicle function. Poor dietary intake, malabsorption issues, or gastrointestinal disorders can lead to B12 deficiency, which may impair hair growth and contribute to increased hair shedding [30].

Furthermore, we observed significantly lower folate levels in women with hair loss compared to the control group in this study. This finding is consistent with previous research reporting reduced folate concentrations in TE group compared to healthy individuals (7.94 ± 8.98 ng/mL vs. 11.31 ± 4.7 ng/mL; *p* = 0.001) [42].

Folate levels may be reduced in hair loss patients because folate is essential for DNA synthesis and cell division in the rapidly proliferating hair follicle cells. A deficiency may impair follicular growth and regeneration, contributing to hair thinning and loss. Poor dietary intake or increased physiological demand may explain the lower levels observed in affected individuals [30].

Similarly, the current study found that the mean serum TSH and FT4 levels in hair loss patients were significantly reduced compared to healthy controls. A similar trend of reduction in mean serum TSH concentration in hair loss patients (2.15 ± 1.41 mIU/mL) was previously reported, compared to (2.11 ± 1.09 mIU/mL) in the control group. However, this difference was not statistically significant [26].

A cross-sectional investigation involving 120 female patients with diffuse hair loss revealed that thyroid function disorders differed significantly between patients with concomitant illnesses and those without, as well as between controls and patients with generalized hair loss [45]. These findings are corroborated with those of current study.

Additionally, Prasad et al. (2023) found that TSH levels in patients with hair loss ranged from 0.379 to 5.078 µIU/L (mean 2.25 µIU/L), whereas the control group ranged from 0.30 to 4.30 µIU/L (mean: 1.78 µIU/L) [46].

On the other hand, Hegde and Noronha (2020) found no significant difference in TSH levels between patients (2.15 ± 1.41 mIU/mL) and healthy controls (2.11 ± 1.09 (mIU/mL), with a *p*-value of 0.877 [26].

The significantly lower mean serum TSH and FREE T4 levels observed in the patient group in the current study may reflect subtle thyroid dysregulation, which can disrupt the normal hair growth cycle. Thyroid hormones play a crucial role in hair follicle development and metabolism, and even mild imbalances can contribute to hair thinning or increased shedding [47].

Enormous and conflicting evidence exists on the role of selenium, zinc, copper, and calcium in hair loss among women. A noteworthy finding in our study is that the means of serum levels of selenium, zinc, copper, and calcium were significantly lower in women with hair loss compared to controls. This finding is consistent with other studies reporting that patients with androgenetic alopecia, particularly men, often show deficiencies in zinc, copper, magnesium, and selenium [48]. It also has been reported that concentrations of zinc were much lower in chronic telogen effluvium (CTE) participants [43]. Another study reported a reduction in serum zinc levels among women with FPHL compared to normal reference ranges [33]. However, our results do not correspond with that study in detected significant increase in selenium level in women with hair loss compared to controls [43].

In agreement with our findings, it has been shown that the predictive efficacy of zinc, copper, and selenium levels in identifying the presence of CTE is found to be statistically significant. It was noted that the copper/zinc ratio and the selenium concentrations were important indicators of CTE [43].

The observed recued levels of serum selenium, zinc, copper, and calcium in women with hair loss in our study may be due to these trace elements are essential for maintaining healthy hair follicle function, keratin production, and antioxidant defense. Deficiencies in these elements can impair hair shaft strength, delay growth, and increase hair shedding due to weakened structural and enzymatic support within the follicles [49].

Although the reasons for discrepancies in the reported findings in this context are not yet clear, they may be attributed to the differences in the study design, sample size, geographical region and participants selection criteria.

In the current study, hair loss was significantly associated with deficiencies in selenium, folate, vitamin B12, vitamin D, and ferritin, as well as lower TSH and younger age.

Nasrin et al. (2013) identified different predictors of hair loss [20]. The multivariate logistic regression modeling revealed that age (CI: 95% 0.672 to 3.714, OR 2.013), family history of hair loss (CI 95% 0.162 to 1.991, OR 1.231), and a decreased blood ferritin content (CI 95% 1.043 to 1.139, OR 1.090) were significantly associated with FPHL. These results align with earlier studies [50,51].

Rasheed et al. (2013) identified the key risk variables for hair loss (TE and FPHL) through multivariate logistic regression analysis [34]. Age, serum ferritin, and vitamin D2 levels were found to be highly predictive of hair loss (*p* = 0.000, OR = 1.175; *p* = 0.013, OR = 0.977; *p* = 0.000, OR = 0.933), respectively.

This study had several notable strengths. This is a prospective case–control study with well-defined inclusion and exclusion criteria, ensuring the validity and homogeneity of the sample. By recruiting age-matched controls and conducting rigorous biochemical and hematological analyses using standardized laboratory techniques and reference ranges, the study enabled robust comparisons between women with hair loss and healthy controls. Furthermore, the study evaluates a broad range of relevant biochemical and hormonal markers at the same time, which provides a more holistic view of potential physiological contributors to hair loss. Moreover, the use of multiple linear regression analysis allowed the identification of independent predictors of hair loss, thereby strengthening the interpretation of associations while adjusting for confounding variables. Findings also could have direct implications for the clinical evaluation and management of women presenting with hair loss.

Our study has some limitations. First, the sample was drawn from private dermatology clinics in a single city, which may limit the generalizability of the findings to the broader Jordanian or regional female population. Second, important confounding factors such as dietary habits, psychosocial stressors, and genetic predispositions—which may significantly influence micronutrient levels and hair health—were not assessed. Furthermore, the cross-sectional nature of the biochemical assessments precludes conclusions about causality. Lastly, although many parameters were analyzed, certain micronutrients and hormonal factors relevant to hair physiology were not included.

## 5. Conclusions

This study demonstrated a statistically significant association between hair loss in females and reduced levels of several hematological and biochemical parameters, including hemoglobin, iron, ferritin, copper, selenium, calcium, vitamin D, and vitamin B12, as well as altered thyroid hormone levels. Multiple linear regression analysis identified hemoglobin, iron, and copper as the most influential predictors of hair loss. These findings suggest that subclinical deficiencies in essential micronutrients and hormonal imbalances may contribute to the pathogenesis of hair loss in females, even when laboratory values fall within the reference range. Although our study focused exclusively on women, it is reasonable to hypothesize that micronutrient deficiencies such as copper, selenium, and folate could also contribute to generalized hair thinning in men, particularly in those misdiagnosed with or refractory to androgenetic alopecia. The potential role of other micronutrients in male patients remains underexplored. Furthermore, several biologically relevant nutrients were not assessed in our study, including magnesium, calcium, essential amino acids, and biotin, all of which have been implicated in hair follicle biology. Future studies should aim to investigate these micronutrients in both female and male populations to clarify their role in hair loss pathophysiology and to guide more comprehensive diagnostic and therapeutic approaches.

Therefore, this study recommends routine screening of key hematological and biochemical parameters should be considered in the diagnostic evaluation of female patients presenting with hair loss. Targeted nutritional interventions and appropriate supplementation may help improve clinical outcomes in affected individuals. Future research into the pathogenesis of hair loss in women should involve larger, multicenter cohorts and consider longitudinal follow-up to better establish causal relationships. Additionally, investigating the impact of dietary intake, genetic predisposition, and psychosocial factors would provide a more comprehensive understanding of the multifactorial nature of female hair loss. Future prospective studies should incorporate variables such as menopausal status, menstrual history, BMI, lifestyle, and supplement use to complement biochemical findings.

## Figures and Tables

**Table 1:** Age distribution between female with hair loss and controls.

Variable	Parameter	Hair Loss Patients *N* = 100	Controls *N* = 100	Test	*p*-Value
Age	Mean ± SD	43.06 ± 10.76	41.39 ± 7.94	χ^2^ test =0.08	0.88
	Min–max	26–65	26–59		
	<45 years	56 (56%)	58 (58%)		
	≥45 years	44 (44%)	42 (42%)		

χ^2^ test = chi square test.

**Table 2:** Comparison of hematological parameters between female with hair loss and controls.

Variable	Parameter	Hair Loss Patients *N* = 100	Controls *N* = 100	Normal Range	f-Value	*p*-Value
Hb (g/dl)	Mean ± SD	11.45 ± 0.39	13.09 ± 0.46	11.6–15	727.71	<0.001
	Min–max	10.70–12.40	11.90–14.10			
Iron (µg/dL)	Mean ± SD	70.14 ± 7.85	94.42 ± 5.61	70–120	632.42	<0.001
	Min–max	54–87	78–113			
Ferritin (ng/mL)	Mean ± SD	39.34 ± 3.71	48.09 ± 5.31	13–150	182.35	<0.001
	Min–max	33–53	39–59			

One-way ANOVA (f-test).

**Table 3:** Comparison of serum trace elements and calcium levels between female with hair loss and controls.

Analyte	Parameter	Hair Loss Patients *N* = 100	Controls *N* = 100	Normal Range	f-Value	*p*-Value
Se (µg/dL)	Mean ± SD	67.11 ± 5.53	71.45 ± 4.05		40.04	<0.001
	Min–max	56–78	63–79			
Zn (µg/L)	Mean ± SD	86.07 ± 3.98	88.87 ± 2.03	70–110	39.12	<0.001
	Min–max	78–95	85–94			
Cu (µg/L)	Mean ± SD	90.71 ± 3.48	104.84 ± 5.38	70–140	489.13	<0.001
	Min–max	83–98	96–119			
Ca (mg/dL)	Mean ± SD	8.61 ± 0.28	9.11 ± 0.27	8.4–10	153.67	<0.001
	Min–max	7.8–9.3	8.5–9.8			

One-way ANOVA (f-test).

**Table 4:** Comparison of thyroid function parameters between female with hair loss and controls.

Variable	Parameter	Hair Loss Patients *N* = 100	Controls *N* = 100	Normal Range	f-Value	*p*-Value
TSH (µIU/mL)	Mean ± SD	1.74 ± 0.25	2.35 ± 0.39	0.4–4.2	169.91	<0.001
	Min–max	1.29–2.24	1.39–3.42			
Free T4 (ng/dL)	Mean ± SD	1.11 ± 0.11	1.32 ± 0.12	0.7–1.7	170.64	<0.001
	Min–max	0.84–1.40	0.99–1.65			

One-way ANOVA (f-test).

**Table 5:** Comparison of serum folate, vitamin B12, and vitamin D levels between female with hair loss and controls.

Variable	Parameter	Hair Loss Patients *N* = 100	Controls *N* = 100	Normal Range	f-Value	*p*-Value
Folate (ng/mL)	Mean ± SD	6.17 ± 0.63	6.96 ± 0.41	3–17	109.58	<0.001
	Min–max	4.8–8.5	5.7–8			
B12 (pg/mL)	Mean ± SD	185.52 ± 35.27	258.30 ± 52.84	160–950	131.21	<0.001
	Min–max	123–267	187–417			
Vit D (ng/dL)	Mean ± SD	26.32 ± 2.98	32.20 ± 3.76	30–100	149.74	<0.001
	Min–max	21–34	25–42			

One-way ANOVA (f-test).

**Table 6:** Analysis of biochemical predictors associated with hair loss in females.

Variable ^a^	Odds Ratio (OR)	95% CI (Lower–Upper)	*p*-Value
Age	0.94	0.89–0.98	0.008
Selenium	0.62	0.38–0.98	0.041
TSH	0.44	0.22–0.90	0.025
FREE T4	0.71	0.36–1.38	0.319
Hb	0.74	0.52–1.05	0.094
Zinc	0.98	0.95–1.01	0.161
Copper	1.01	0.98–1.04	0.401
Folate	0.79	0.63–0.99	0.045
Vitamin B12	0.99	0.98–0.99	0.012
Vitamin D	0.94	0.89–0.99	0.021
Calcium	0.88	0.67–1.16	0.362
Iron	0.98	0.96–1.00	0.076
Ferritin	0.96	0.93–0.99	0.033

^a^ Dependent Variable: Presence of hair loss. Abbreviations: TSH = Thyroid-stimulating hormone; T4 = Thyroxine; Hb = Hemoglobin; OR = Odds ratio; CI = Confidence interval. Statistical test: Multivariable binary logistic regression.

## Data Availability

The datasets generated during and/or analyzed during the current study are available from the corresponding author on reasonable request.
